# Lens densitometry for assessment and prediction of cataract progression after pars plana vitrectomy with C3F8-gas for retinal detachment

**DOI:** 10.1371/journal.pone.0254370

**Published:** 2021-07-12

**Authors:** Philipp Schindler, Luca Mautone, Eileen Bigdon, Vasyl Druchkiv, Martin Stefan Spitzer, Christos Skevas

**Affiliations:** 1 Department of Ophthalmology, University Medical Center Hamburg-Eppendorf, Hamburg, Germany; 2 Department of Research & Development, Clínica Baviera, Valencia, Spain; Massachusetts Eye & Ear Infirmary, Harvard Medical School, UNITED STATES

## Abstract

**Purpose:**

Lens opacification is a common complication after pars plana vitrectomy (PPV) and knowing its progression would facilitate consulting patients. The purpose of this study was to evaluate a quantitative model for lens-status-monitoring after PPV with C3F8 gas. Our model was evaluated in rhegmatogenous retinal detachment (RRD) patients of various age and lens densitometry (LD).

**Methods:**

Data between March 2018 and March 2020 were evaluated retrospectively. LD measurements of the PentacamHR® Nucleus Staging mode (PNS) were used to quantify lens opacification over time. A mixed-effect regression model was designed, to enable LD predictions at any time postoperatively. Calculations were based on patient’s age and baseline LD as dependent variables. Six patients were randomly excluded during model development, to be used for testing its power afterwards.

**Results:**

34 patients (male 19 [55.9%], female 15 [44.1%]) matched the inclusion criteria. Average age was 58.5 years (32–77;±4.3) and average follow-up was 7.2 months (3,4–23.1;±1,8). Mean baseline LD of the treated and fellow eye before surgery were 10.9% (8.7%-14.8%;±0.8) and 10.7% (8.5%-14.1%;±0.6), respectively. Using our prediction model, LD values for the six pre-selected patients closely match the observed data with an average deviation of 1.07%.

**Conclusions:**

Evaluation of age and baseline LD using a mixed-effect regression model might predict cataract progression in RRD patients treated with PPV and C3F8-gas. Such a tool could be considered during cataract surgery consultation in these patients.

## Introduction

Lens opacification is a common complication following pars plans vitrectomy (PPV). Combined cataract correction and PPV is more frequently performed in patients over 50 years of age, but it may also be considered in younger patients. In a recent study of 180 patients, 72–96% developed sight-impairing lens opacification within 24 months after PPV [1].

Increased intravitreal oxygen during the procedure, and subsequent free radical formation might comprise the main inciting event. Particularly in the retrolental space, aggregation of such radicals and denatured protein fragments are considered to potentiate cataract progression [2, 3].

In previous studies, post-PPV cataract surgery was required for 63.2% of the cases originally operated for vitreomacular traction, 80% of macular pucker repairs, and 64.6% of macular hole surgeries [4–6]. Clinically significant opacification occurred within 6 months after macular hole surgery, depending on the use of adjunctive intravitreal gas tamponade. Using intravitreal gas instead of balanced salt solution will increase the risk of developing nuclear sclerosis by approximately 60% [7–9]. To date the kinetics, degree and predictability of cataract development after PPV have not been precisely described.

The aim of this study was to quantify and predict the effect of PPV with C3F8 (16% perfluoropropane) gas on the lens status. For this purpose, we evaluated patients of various age and lens densitometry values that were treated for rhegmatogenous retinal detachment (RRD) via the aforementioned regimen. For quantification, the PentacamHR® Nucleus Staging (PNS) module of the Scheimpflug tomography system (Oculus, Wetzlar, Germany) was used.

## Methods

Our database was retrospectively searched for patients with RRD treated with 23G PPV using C3F8 (16% perfluoropropane) gas between March 2018 to March 2020. The study followed the tenets of the Declaration of Helsinki and was approved by the Medical Institutional Review Board of Hamburg (PV7250). Patient’s data were gathered fully anonymized.

Our exclusion criteria included cataract surgery (including those performed simultaneously with PPV), PPV, or any other intraocular procedure. Also, patients were excluded if they had history of ocular trauma, uveitis, topical or systemic corticosteroid therapy, and signs of visually-impairing cataract at baseline evaluation. In terms of past medical history, we did not include patients with known topical or systemic conditions that could accelerate cataract formation and/or progression after PPV. Patients with ischemic and/or proliferative retinopathy were also excluded [10–12].

All patients underwent a complete ophthalmological examination, including BCVA, intraocular pressure measurement, anterior segment examination with slit lamp biomicroscopy, dilated fundoscopy, and PentacamHR® lens densitometry prior to surgery and at every visit during follow-up. Post-operative follow-up visits were scheduled at about six weeks, three months, and six months based on our clinic’s protocol for RRD patients.

**Fig 1:**
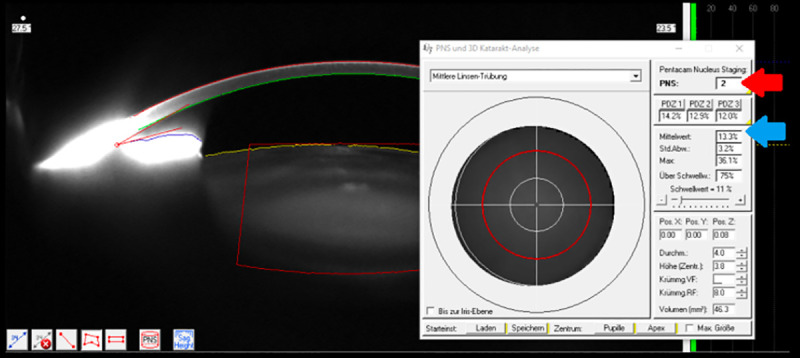
PentacamHR® Nucleus Staging (PNS) module. PentacamHR® Nucleus Staging (PNS) module showing a Scheimpflug cross-sectional image of the anterior eye segment and lens densitometry with PNS value (red arrow) and mean lens densitometry (blue arrow).

PentacamHR® measurements were taken under standard dim-light conditions and after pupil dilation. Mean lens densitometry (LD) value were calculated by the PNS module in predefined three-dimensional volumes centered on the apex. This system reveals a three-dimensional image of the crystalline lens, and delivers LD parameters in the chosen area as percentages: 0% means no backward scatter, and 100% means total backward scatter of the lens. Included PNS ranges from 0 (no cataract) to 5 (progressed cataract), as shown in Fig 1.

Distorted PentacamHR® images due to high reflection were considered non-eligible for evaluation. This method has been approved by various studies in advance [13–18].

There were no cases of intra-operative lens touch, or similar complications increasing the risk of cataract formation. Patients who had their fellow eye operated during follow-up, as well as those who needed revision surgery to address a complication were excluded from the study. If cataract surgery was required during the investigation period, it was marked as the endpoint of that particular case. All parameters were also evaluated on the fellow, non-treated eye; the latter also provided reference values for LD per case. To ensure that LD was always taken from the same 3D-volume of the crystalline lens, each volume was aligned by hand; then it was stored, to be used for all follow-up measurements in the same patient. We selected the following variables to coevaluate with post-surgical LD changes: time (months), baseline LD (%), age at the time of surgery (years).

## Results

34 eyes of 34 patients matched the inclusion criteria. Demographic and presurgical parameter are summarized in Table 1. One patient suffered from postsurgical macular edema and optic atrophy most probably due to PPV as a complication. No entry has been found in the patient’s file which might have led to this condition without surgery. So, this case did not lead to an exclusion from the study.

**Table 1:** Demographics and presurgical parameter.

**Patients**	Total n = 34
**Right eye**	n = 16 (47.1%)
**Left eye**	n = 18 (52.9%)
**Female**	n = 15 (44.1%)
**Male**	n = 19 (55.9%)
**Age (years)**	32–77; 58.5 (±4.3)
**BCVA (LogMAR)**	0.0–1.0; 0.4 (±0.3)
**Lens Densitometry baseline treated eye (%)**	8.7–14.8; 10.9 (±0.8)
**Lens Densitometry baseline fellow eye (%)**	8.5–14.1; 10.7 (±0.6)
**Diabetes Type I**	n = 1 (2.9%)
**Diabetes Type II**	n = 5 (14.7%)
**Follow-up time (months)**	3.4–23.1; 7.2 (±1.8)

Qualitative parameters are presented as percentages, and quantitative ones as min to max and mean (±Standard deviation).

Gender and side of surgery were almost equally distributed (male 19 [55.9%], female 15 [44.1%], right eye 16 [47.1%], left eye 18 [52.9%]). Patient’s age ranged from 32 years to 77 years with an average of 58.5 (±4.3) years. Mean baseline LD of the treated and fellow eye were 10.9% (8.7%-14.8%;±0.8) and 10.7% (8.5%-14.1%;±0.6), respectively.

Average follow-up was 7.2 months (3.4–23.1; ±1.8). Six patients suffered from diabetes; one of these had type I diabetes, the rest had type II. None of these patients was excluded from the study, since they had no signs of diabetic retinopathy (ischemic or non-ischemic) during the period of investigation.

### Statistics and prediction model

To ensure that the model can be applied for a prediction of new cases, we took out 6 random eyes from the initial database of 34 eyes for testing the model, while the remaining 28 eyes were used to create and train the model.

**Fig 2:**
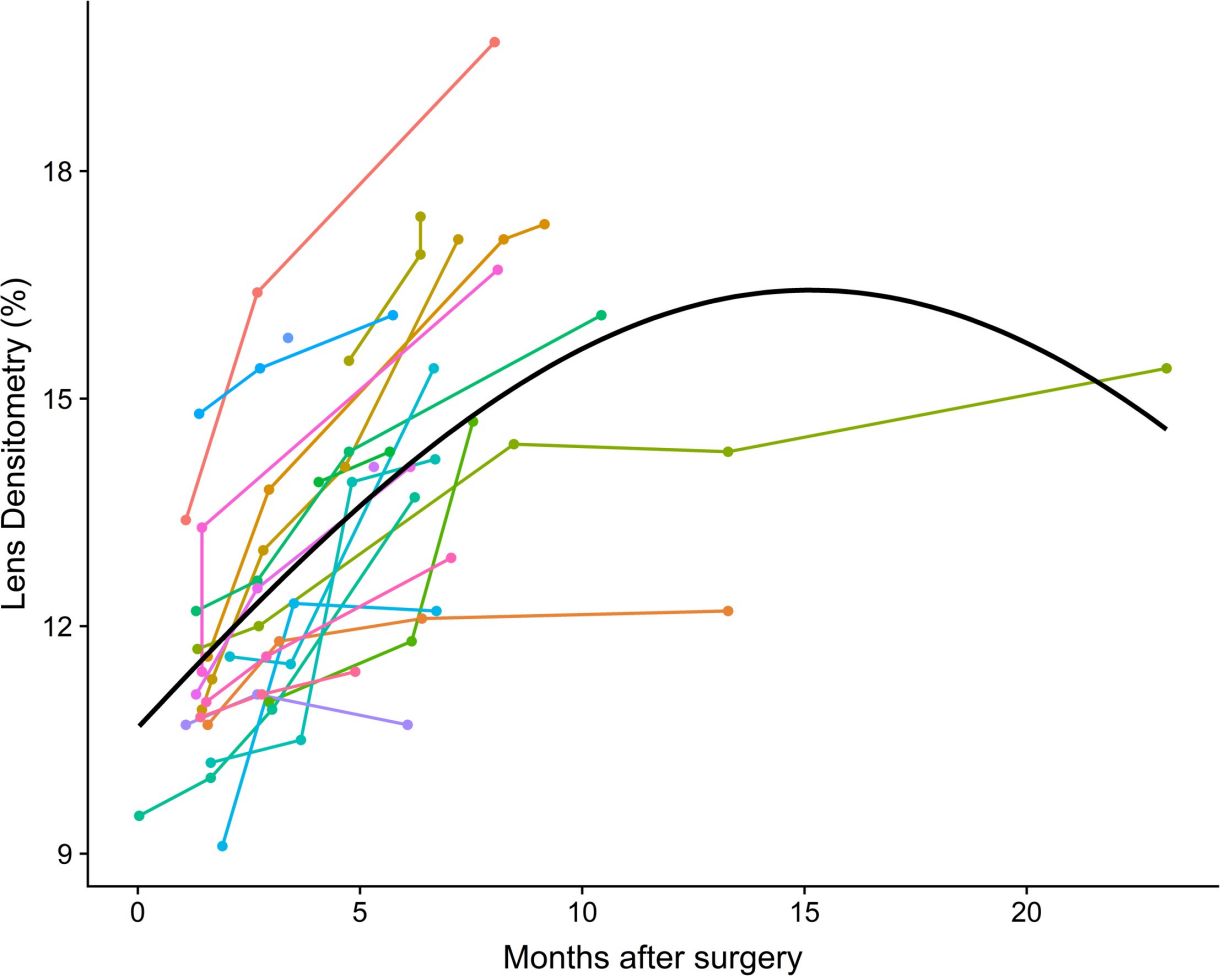
Trajectories of lens densitometry changes. Trajectories of lens densitometry changes (observed post-surgical measurements) for treated eyes.

Fig 2 shows the observed post-surgical LD trajectories of the remaining 28 treated eyes. We can see steeper curves of LD especially during the first months after surgery. After 3–4 months this effect seems to get weaker. But steep increase of LD is not always the case as one can see from some trajectories having a rather flat course.

After testing various mathematical and statistical models we decided that a mixed effects regression model showed most promising results for a prediction.

For the calculations we set a maximum of 100% LD to limit the prediction model, because 100% is the possible maximum of the PNS module (meaning all incoming light is scattered backwards from the lens). Additionally, we found out that a limit of 100% will also produce a better natural behaviour of the mathematical model and bring forth best fit predictions. In clinical everyday life lens opacification will of course not reach an unusually high LD of 100%, because vision impairment would be substantially and will preferably lead to cataract surgery, soon. Additionally, clinical observations are unlikely to be made for any longer time when vision impairment is already present. That is why none of our patients exceeded a value of 20% as one can tell when looking at the results.

The supplement document of this paper gives further information on statistical calculations, prediction of LD, selected trajectories and results of the fellow eyes, as well as the standard surgical technique. In general, we used backwards variable selection algorithm beginning with the saturated model (all interactions included). Table 2 shows a summary of the mathematical results of the mixed effects regression model.

**Table 2:** Mixed effects regression model results.

	Dependent variable: $\frac{\frac{LD}{100}}{1-\frac{LD}{100}}$
**Months**	-0.0125 (0.0212)
**Months^2^**	-0.0025*** (0.0004)
**Baseline lens densitometry**	0.0693*** (0.0148)
**Age at surgery (years)**	0.0011 (0.0029)
**Months x age**	0.0014*** (0.0004)
**Intercept**	-2.9965*** (0.1798)
**Observations**	98
**Log Likelihood**	88.3736
**Akaike Inf. Crit.**	-160.7473
**Bayesian Inf. Crit.**	-140.0675

P-Values are given symbolically with asterisks:

*p<0.1

**p<0.05

***p<0.01. In parenthesis are standard errors of the coefficients.

The model delivers a set of coefficients that describe the interaction between dependent factors, for example “months after surgery” or “baseline LD”, and the prediction. The selection of the factors has been done according to the results of the statistical analysis, depending on whether they have significant influence on lens opacification or not. In this way we received the following results:

There is a significant effect of time (months after surgery). But the effect of time enters the model in three ways: as a linear effect, non-linear effect and as an interaction with age. That explains why “months” appears several times and in different ways in our equation further below.

Furthermore, baseline LD is positively related to postoperative LD, meaning that the higher baseline LD is, the higher the predicted trajectories will be.

The effect of age is also significant, but not as much as baseline LD. Therefore, baseline LD is a higher influential cofactor than age. In the model we consider age as continuous variable. All these findings were considered when calculating the coefficients. Using the results we came up with the following prediction equation:

$$\begin{array}{l} \mathrm{Logit}(LD)=-2.9965\\ \;-0.0125\times \mathrm{Months}-0.0025\times {\mathrm{Months}}^{2}\\ \;+0.0693\times \mathrm{Baseline}\;\mathrm{LD}\\ \;+0.0011\times \mathrm{Age}\\ \;+0.0014\times \mathrm{Months}\times \mathrm{Age} \end{array}$$

Where,

$$Logit(LD)=\frac{\frac{LD}{100}}{1-\frac{LD}{100}}$$

To get the final prediction we have to back-transform from the logit scale as follows:

$$LD=100\times \frac{e^{Logit(LD)}}{1+e^{Logit(LD)}}$$

By filling the equation with individual values (“months” after surgery, “baseline LD” and “Age” of the Patient) one can calculate an estimated LD for any time after surgery for any patient facing a PPV with C3F8-gas due to RRD.

### Model performance

To assess the performance of the predictive model we now use these six patients that had been excluded in advance for validation purpose.

**Fig 3:**
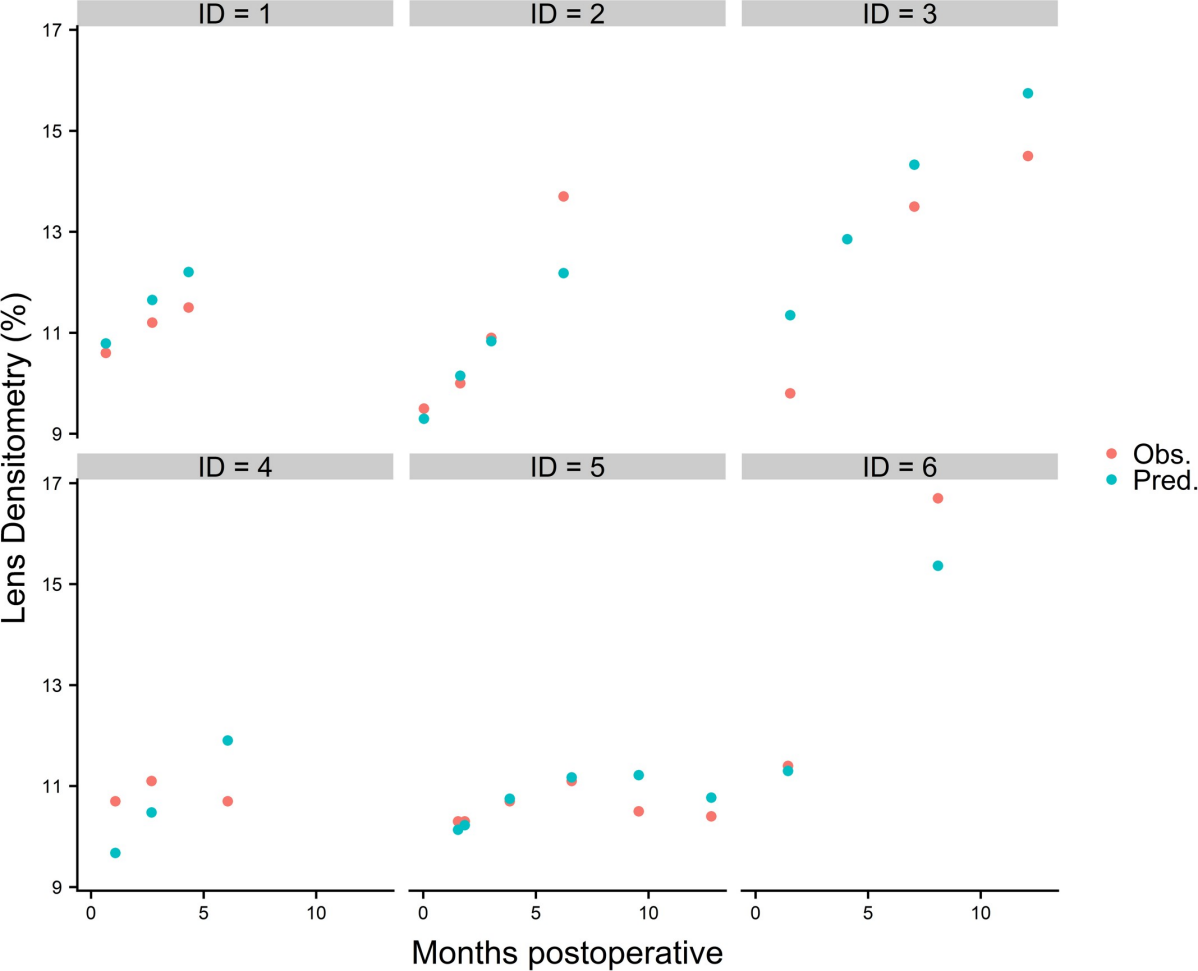
Predicted and observed lens densitometries over time. Predicted (blue dots) and observed (red dots) postoperative lens densitometries over time.

In Fig 3 blue dots show predicted LD values from the model. Red dots depict actual LD values from the patient’s PentacamHR® file, respectively.

Apart from ID 4, predicted LD values of the individuals (ID) closely match the observed data in the patient’s file. The mean deviation from the observed data stretched from 0.34% to 1.62% (average 1.07%). Finally, one can see that the prediction model estimates LD in a clinically acceptable way to our opinion. However, it must be mentioned that due to the low number of cases especially for the period after 12 months postoperatively, the performance of the model is limited.

### Web application for predicting postoperative lens densitometries

We developed a web application that calculates the individual postoperative trajectories for LD based on the model shown above. The user must provide two parameters only: age in years and baseline LD. The application makes all background calculations and displays results as a line plot. Furthermore, age intervals and baseline LD ranges of our dataset can be visualized, too. The application can be accessed free under the following link: https://statisticarium.com/apps/sample-apps/PredictLensDensity/.

## Discussion

Despite the low number of patients in this study, one can see that both values, the observed and the predicted ones, of six previously excluded individuals are close together in most of the cases. There are deviations in the prediction, yet, which becomes especially apparent when looking at the trajectories of ID 4 (Fig 3). The model estimates a steep increase of LD starting at about 8,5% at six weeks after surgery and reaching 12% after six months. However, observed data showed a rather flat curve at around 10.5–11.0%. Presumably, the accuracy of the prediction will improve when more data is used to train the model.

Lens opacification is a common complication after PPV, despite the technical evolution of the surgical method over the years. The exact etiology of cataract formation after PPV is unknown, but it may relate to increased oxygen levels and the ensuing oxidative stress, altered milieu following vitreous removal, trauma, and iatrogenic causes [2–3].

The studies of Palmquist and Filas showed that oxidative stress by hyperbaric oxygen increases the exposure to molecular oxygen and can cause human lens opacification.

Seven out of 15 patients, most of them over 50 years of age, treated with hyperbaric oxygen (100% O2 at 2–3 atm) daily for ulcers secondary to peripheral vascular disease developed nuclear opacities after 150 to 850 treatments. None of the control patients developed any signs of lens opacification during the study. The only patient who did not develop symptoms of NSC was 24 years old. This is consistent with observations after PPV that younger patients are less susceptible to post-vitrectomy cataract [19–21].

Filas et al. developed a model which could estimate lens opacification after increase of retrolental oxygen pressure. According to this model, an eye with an intact vitreous body and no posterior vitreous detachment (PVD) will have retrolental partial pressure of oxygen (pO2) levels ranging from 4 to 6 mmHg, whereas an eye that has undergone PPV with PVD induction will have significantly higher levels, with pO2 ranging from 10 to 12 mmHg [19–22].

### Lens opacity classification systems

Various lens opacity grading systems have been developed for grading cataracts. The Lens Opacity Classification Systems II and III (LOCS II and III) use color lens photograph standards or slit-lamp photographs of the lens to quantify its opacity [23].

Scheimpflug cataract imaging method is an objective approach to cataract grading. The developments of the Scheimpflug topography system have enabled quantitative measurements of the lens transparency. Using a rotating Scheimpflug camera PentacamHR® provides precise, three dimensional images of the lens. By producing reproducible data related to lens densitometry, that is a quantitative and objective method to assess the lens transparency [14–16, 24–26].

By applying the LOCS II lens grading scale Thompson et al. showed an increase in nuclear sclerosis in vitrectomized eyes compared with fellow eyes. This increase was about sixfold to sevenfold in all patient age ranges after PPV [20].

Ibáñez-Ruiz et al. found a significantly higher linear optical densitometry utilizing the Pentacam® Scheimpflug camera in 81 vitrectomized phakic eyes when compared to the non-vitrectomized eyes [27].

Weiner et al reported that in the early stages of cataract formation it is difficult to differentiate by using clinical classifications such as LOCS II, LOCS III. The use of lens densitometry analysis like the PentacamHR® Scheimpflug imaging system was preferred because it is precise, objective, and reproducible [16].

The PentacamHR® Scheimpflug method used in our study enabled us to receive not only the baseline preoperative LD on both eyes, but also we were able to document the change in LD in the follow up period precisely (Table 1 and Fig 2). Weakness of the PentacamHR® PNS module is that it only depicts nuclear and no cortical or posterior subcapsular changes.

### Effect of age and baseline Lens Densitometry (LD) on the progression of lens opacification

Age plays an important role in causing postsurgical lens opacification and many surgeons use the patients age to decide whether to perform combined cataract and PPV or not. A combined PPV is performed more frequently in patients >50 years of age but it is sometimes needed in younger patients, too. Melberg et al. observed no significant progression in NSC after PPV in patients <50 years of age compared with patients >50 years of age [28].

Kataria et al. and Thompson et al, suggest that NSC progresses at a rate 6- to 9-fold higher in patients >50 years of age compared with patients <50 years of age. This finding suggests that cataract surgery should not be performed routinely combined with PPV in patients less than 50 years of age because cataract surgery is usually not needed for many years [12, 20, 29].

There is still no consensus about whether combined cataract and PPV surgery should be generally performed for patients over 50 years of age. It is an individual decision of the surgeon based on several factors like clinical status of the lens, level of difficulty of the planned PPV, preferences of the patient and skill of the surgeon.

Our study could clearly depict that the lens densitometry trajectory in younger patients is flat meaning that the change in lens opacification is slow and not very intense in the months following PPV compared to older patients. However, when using our model, more precise predictions were obtained by giving baseline LD a higher influence in the equation than age of the patient. This method is supported by the fact that baseline LD of older patients is also higher according to the natural behavior of the lens.

In our model the effect of age and the interaction with time after surgery are significant.

Older patients tend to develop faster (steep trajectories) and denser lens opacification than younger patients (flatter trajectories). Younger age seems to protect the lens from lens opacification because not only is the rate of lens opacification slower, but also the postsurgical LD shows less to almost no change in younger patients for at least 6 months after PPV.

So, what we see as highly promising about this approach is the fact that baseline LD has a greater influence on the prediction model than age. Furthermore, not age alone will be taken into the equation, but also its interaction with baseline LD. This might be the cause why the prediction of the model is so accurate.

Using a top-limit prevents the model from predicting unnaturally high LDs and seems to fit the natural course of lens opacification better.

These findings are matching clinical observations and scientific results dealing with this topic.

## Conclusions

PPV leads to more rapid oxidative damage of the crystalline lens and thus promotes significant progression of lens opacification. A patient who is about to undergo PPV should always be informed about the inevitable ensuing lens opacification. Consultation on the possibility to combine PPV with cataract surgery is required in certain cases. Up until now cataract surgery was mostly performed in patients >50 years, but this conclusion was based on parameters that could be subjected to bias, like empirical observation, changes in the post-surgical refraction and slitlamp examination of the lens. Lens Opacity Classification System II+III (LOCS II+III) may also help. The use of PentacamHR® for the measurement of lens opacification excludes the danger of objective bias by the surgeon or the investigator. Limitations of this study included the absence of long-term follow-up period in many eyes. A longer follow-up would allow a more precise regression model to better characterize cataract progression with elapsed time after surgery not only in the operated eye but also in the fellow eye. In our study no significant changes in LD in the fellow eye was observed. To our knowledge this is the first study to document and predict the effect of 23G PPV and C3F8-gas endotamponade on patients suffering from RRD and excluding parameters that could have an extra effect on the lens opacification (e.g. re-PPV after re-detachment, diabetic retinopathy, systemic cataractogenic conditions like steroid intake etc.). Using an objective parameter like LD additionally to the patient’s age has led to very promising results in our prediction model. The accuracy of the model was especially obtained by giving baseline LD a higher influential cofactor in the equation than patient’s age. The application on the of lens opacification (which can be accessed free under the following link https://statisticarium.com/apps/sample-apps/PredictLensDensity/) can help vitreoretinal surgeons reaching a safer and more reliable conclusion on the rate of postsurgical lens opacification just by importing baseline LD prior to surgery and age of the patient. This tool could be proved valuable during the preoperative discussion with patients.

We hope these results could enrich the data on cataractogenesis after PPV and help vitreoretinal surgeons to better counsel patients and plan or avoid future surgeries.

## Supporting information

S1 File (DOCX)
