# Supplementary material for: Lens densitometry for assessment and prediction of cataract progression after pars plana vitrectomy with C3F8-gas for retinal detachment
Source: PLoS One. 2021 Jul 12;16(7):e0254370. doi: 10.1371/journal.pone.0254370 (PMC8274854; doi:10.1371/journal.pone.0254370)
Supplement: S1 File — (DOCX) [file pone.0254370.s001.docx]

S1 File

Lens densitometry for assessment and prediction of cataract progression after pars plana vitrectomy with C3F8-gas for retinal detachment

Philipp Schindler MD^1^*^¶^, Luca Mautone MD^1¶^, Eileen Bigdon MD^1^, Vasyl Druchkiv ^2^, Martin Stefan Spitzer MD^1^, Christos Skevas MD^1^

^1^Department of Ophthalmology, University Medical Center Hamburg-Eppendorf, Hamburg, Germany

^2^Department of Research & Development, Clínica Baviera, Valencia, Spain

^¶^These Authors contributed equally to the work

***Corresponding author:**

Dr. med. Philipp Schindler, FEBO

Email: [p.schindler@uke.de](mailto:p.schindler@uke.de) (PS)

# Surgical technique

Surgeries were performed under local or general anesthesia according to the preferences of the patient. The same surgical method was used in all patients (23gauge vitrectomy). Trocars were inserted in the inferotemporal, superotemporal, and superonasal quadrants 3.0–4 mm posterior to the limbus. All eyes underwent a complete vitrectomy (scleral depression performed in all patients), followed by fluid-air exchange and endolaser treatment to re-attach and fixate the retina. A final air – gas (16% perfluoropropane (C3F8)) exchange was performed. At the end of the surgery, microcannulas were removed from the eye. No sutures were needed to close the scleral or conjunctival openings. No intraoperative complications or lens touch occurred in any eye.

# Predicting lens densitometry

## Prediction equation

*Logit(LD)* = − 2.9965

− 0.0125 × Months − 0.0025 × Months^2^

+ 0.0693 × Baseline LD

+ 0.0011 × Age

+ 0.0014 × Months × Age

Where,

$$Logit\left( LD \right)= \frac{\frac{LD}{100}}{1- \frac{LD}{100}}$$

To get the final prediction the logit scale was back-transformed as follows:

$$LD=100 \times\frac{e^{Logit(LD)}}{1+ e^{Logit(LD)}}$$

## Examples

Let us apply this formula for predicting of postoperative LD at 1 month, 3 months, 6 months and 12 months after surgery:

**S1 Table. Example patient 1.**

|  | **Patient 1**  Baseline LD = 9; Age = 40a | | | | |
| --- | --- | --- | --- | --- | --- |
|  | *1 month* | *3 months* | *6 months* | *12 months* |  |
|  | Logit(LD) =  -2.9965 − 0.0125 × 1  -0.0025 × 1^2^  +0.0693 × 9 + 0.0011 × 40  +0.0014 × 1 × 40  = -2.289 | Logit(LD) =  -2.9965 − 0.0125 × 3  -0.0025 × 3^2^  +0.0693 × 9 + 0.0011 × 40  +0.0014 × 3 × 40  = -2.222 | Logit(LD) =  -2.9965 – 0.0125 × 6  -0.0025 × 6^2^  +0.0693 × 9 + 0.0011 × 40  +0.0014 × 6 × 40  = -2.16 | Logit(LD) =  -2.9965 – 0.0125 × 12  -0.0025 × 12^2^  +0.0693 × 9 + 0.0011 × 40  +0.0014 × 12 × 40  = -2.17 |  |
| Converting  logits to LD | $LD=100 \times\frac{e^{-2.289}}{1+ e^{-2.289}}$ | $LD=100 \times\frac{e^{-2.222}}{1+ e^{-2.222}}$ | $LD=100 \times\frac{e^{-2.16}}{1+ e^{-2.16}}$ | $LD=100 \times\frac{e^{-2.17}}{1+ e^{-2.17}}$ |  |
| LD (%) | **= 9.201** | **= 9.775** | **= 10.341** | **= 10.244** |  |

To show just the effect of age let us repeat the above calculations but taking 80 years as an age input.

**S2 Table. Example patient 2.**

|  | **Patient 2**  Baseline LD = 9; Age = 80a | | | | |
| --- | --- | --- | --- | --- | --- |
|  | *1 month* | *3 months* | *6 months* | *12 months* |  |
|  | Logit(LD) =  -2.9965 − 0.0125 × 1  -0.0025 × 1^2^  +0.0693 × 9 + 0.0011 × 80  +0.0014 × 1 × 80  = -2.191 | Logit(LD) =  -2.9965 − 0.0125 × 3  -0.0025 × 3^2^  +0.0693 × 9 + 0.0011 × 80  +0.0014 × 3 × 80  = -2.012 | Logit(LD) =  -2.9965 − 0.0125 × 6  -0.0025 × 6^2^  +0.0693 × 9 + 0.0011 × 80  +0.0014 × 6 × 80  = -1.781 | Logit(LD) =  -2.9965 − 0.0125 × 12  -0.0025 × 12^2^  +0.0693 × 9 + 0.0011 × 80  +0.0014 × 12 × 80  = -1.455 |  |
| Converting  logits to LD | $LD=100 \times\frac{e^{-2.191}}{1+ e^{-2.191}}$ | $LD=100 \times\frac{e^{-2.012}}{1+ e^{-2.012}}$ | $LD=100 \times\frac{e^{-1.781}}{1+ e^{-1.781}}$ | $LD=100 \times\frac{e^{-1.455}}{1+ e^{-1.455}}$ |  |
| LD (%) | **= 10.059** | **= 11.798** | **= 14.419** | **= 18.919** |  |

Finally, we can see a major difference in increase of LD prediction over time between Patient 1 (40 years of age, S1 Table) and Patient 2 (80 years of age, S2 Table) when baseline LD is the same (=9%).

In the same manner we can demonstrate just the effect of baseline LD within the model when choosing two patients of the same age, but with different baseline LD, for example as demonstrated in S3 and S4 Table:

**S3 Table. Example patient 3.**

|  | **Patient 3**  Baseline LD = 9; Age = 50a | | | | |
| --- | --- | --- | --- | --- | --- |
|  | *1 month* | *3 months* | *6 months* | *12 months* |  |
|  |  |  |  |  |  |
| LD (%) | **= 9.409** | **= 10.25** | **= 11.253** | **= 12.008** |  |

**S4 Table. Example patient 4.**

|  | **Patient 4**  Baseline LD = 12; Age = 50a | | | | |
| --- | --- | --- | --- | --- | --- |
|  | *1 month* | *3 months* | *6 months* | *12 months* |  |
|  |  |  |  |  |  |
| LD (%) | **= 11.337** | **= 12.326** | **= 13.502** | **= 14.383** |  |

# Selected trajectories

The above calculations can be done for any time and not only for 1, 3, 6 and 12 months. In S1 Fig. estimated postoperative LDs for 12 types of patients with different combinations of age and baseline LD have been visualized for a continuous time course. Note that the background calculation is the same as in the above section. Only input values for age, baseline LD and time have been altered.


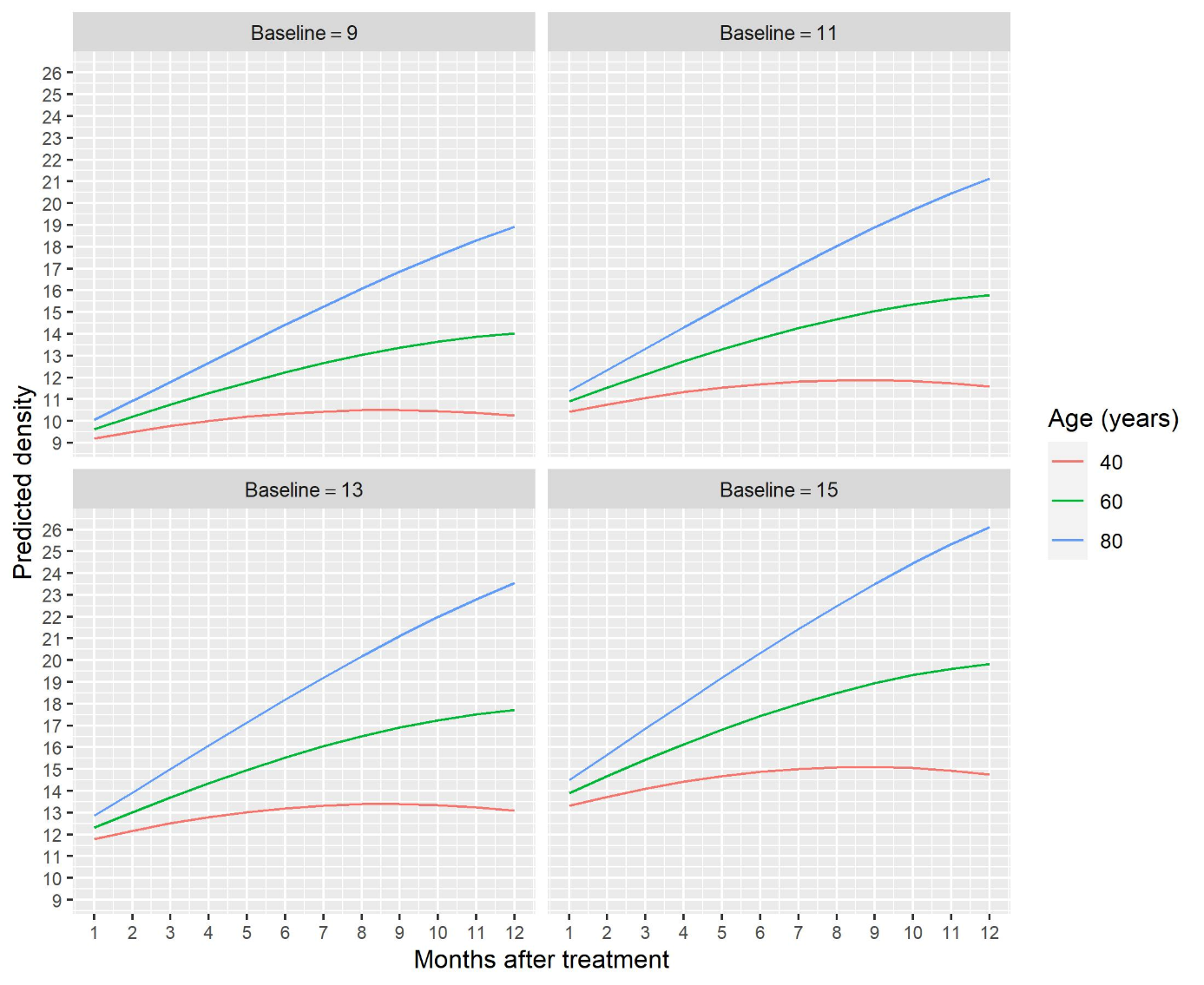


**S1 Fig. Predicted lens densitometries for a continuous time course at three different ages.**

# Results for the fellow eyes

There is no clear pattern in the LD trajectories for the fellow-eyes. Each trajectory seems to run randomly along the timeline. But looking at the course of mean LD of the treated eye and the fellow-eye in the time-course a clear difference can be seen (S2 Fig.). Finally, no significant effect of time or age on LD change during the investigated time could be found for the fellow-eyes.


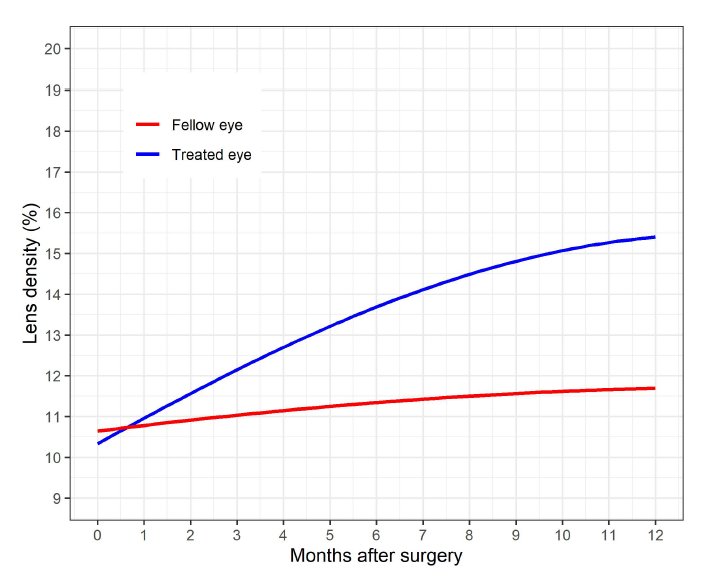


**S2 Fig. Mean lens densitometry of all treated and fellow eyes.**

Results of the mixed effects regression model for fellow eyes is shown in S5 Table for the sake of completeness.

**S5 Table. Mixed effects regression model of fellow eyes.**

|  | Dependent variable:  $\frac{\frac{\boldsymbol{LD}}{\boldsymbol{100}}}{\boldsymbol{1-}\frac{\boldsymbol{LD}}{\boldsymbol{100}}}$ |
| --- | --- |
| Months | -0.0078 (0.0180) |
| Months^2^ | -0.0005 (0.0003) |
| Baseline lens densitometry | 0.0604*** (0.0144) |
| Age at surgery (years) | 0.0026 (0.0025) |
| Months x age | 0.0004 (0.0003) |
| Intercept | -2.9428*** (0.1600) |
| Observations | 99 |
| Log Likelihood | 103.7890 |
| Akaike Inf. Crit. | -191.5779 |
| Bayesian Inf. Crit. | -170.8170 |

P-Values are given symbolically with asterisks: *p<0.1; **p<0.05; ***p<0.01. In parenthesis are standard errors of the coefficients.

# Model accuracy and detailed list of previously selected individuals

S6 Table shows the observed and predicted data of our six previously excluded random individuals. Their age lies between 39 and 64 (average 52.8) and baseline LD between 9.2% and 11.6% (average 10.4%). Compared to average age (58.5) and baseline LD (10.9%) of the other patients the individuals are slightly younger, but start with almost the same baseline LD. Therefore, the randomly selected sample of individuals for testing the model can be considered appropriate.

As far as accuracy of the prediction for these six individuals is concerned the mean deviation from the observed data stretched from 0.34% to 1.62% (average 1.07%). (S6 Table).

The closer deviation reaches zero percent the more accurate is the prediction. Our prediction model reached the highest accuracy for the youngest individual (39 years) with a moderate baseline LD of 10.3%. The worst accuracy was measured at individual 4 (age=55; baseline LD 9.2%). No clear pattern is visible that could explain the differences in accuracy, so further investigation must be made to unravel other possible factors that might influence the dynamics of lens opacification after PPV in cases of retinal detachment.

**S6 Table. Previously selected six individuals and related data of observed and predicted lens densitometries.**

| ID | Age | Baseline  LD in % | Month after surgery | Observed  LD in % | Predicted  LD in % | Deviation =  $\frac{\mathbf{Difference Obs.-Pred.}}{\mathbf{number of measurements}}$ |
| --- | --- | --- | --- | --- | --- | --- |
| 1 | 45 | 11,6 |  |  |  | 1,25 |
| 1 |  |  | 0.66 | 10.6 | 10.79 |  |
| 1 |  |  | 2.72 | 11.2 | 12.52 |  |
| 1 |  |  | 4.33 | 11.5 | 13.17 |  |
| 2 | 55 | 9.5 |  |  |  | 1,14 |
| 2 |  |  | 0.03 | 9.5 | 7.79 |  |
| 2 |  |  | 1.64 | 10.0 | 9.22 |  |
| 2 |  |  | 3.02 | 10.9 | 10.30 |  |
| 2 |  |  | 6.23 | 13.7 | 12.23 |  |
| 3 | 59 | 11.2 |  |  |  | 1,23 |
| 3 |  |  | 1.54 | 9.8 | 11.24 |  |
| 3 |  |  | 4.07 | 12.7 | 13.29 |  |
| 3 |  |  | 7.05 | 13.5 | 14.86 |  |
| 3 |  |  | 12.10 | 14.5 | 16.01 |  |
| 4 | 55 | 9.2 |  |  |  | 1,62 |
| 4 |  |  | 1.08 | 10.7 | 8.36 |  |
| 4 |  |  | 2.69 | 11.1 | 9.67 |  |
| 4 |  |  | 6.07 | 10.7 | 11.78 |  |
| 5 | 39 | 10.3 |  |  |  | 0,34 |
| 5 |  |  | 1.54 | 10.3 | 10.33 |  |
| 5 |  |  | 1.84 | 10.3 | 10.46 |  |
| 5 |  |  | 3.84 | 10.7 | 11.15 |  |
| 5 |  |  | 6.59 | 11.1 | 11.58 |  |
| 5 |  |  | 9.57 | 10.5 | 11.39 |  |
| 5 |  |  | 12.79 | 10.4 | 10.40 |  |
| 6 | 64 | 11.0 |  |  |  | 0,83 |
| 6 |  |  | 1.44 | 11.4 | 10.83 |  |
| 6 |  |  | 8.10 | 16.7 | 15.61 |  |
